# Covert use of reversible contraceptive methods and its association with husband’s egalitarian gender attitude in India

**DOI:** 10.1186/s12889-022-12882-x

**Published:** 2022-03-07

**Authors:** Minakshi Vishwakarma, Chander Shekhar

**Affiliations:** 1 Research Scholar, Department of Fertility and Social Demography, International Institute for Population Sciences, Mumbai, India 400088; 2 Professor, Department of Fertility and Social Demography, International Institute for Population Sciences, Mumbai, India 400088

**Keywords:** Covert contraceptive use, Family planning, Gender attitude, Couples, India

## Abstract

**Background:**

In a patriarchal society, women often keep their use of contraceptives secret in order to meet their reproductive goals and satisfy their reproductive preferences. Nevertheless, to our knowledge, women’s covert contraceptive use and its association with husband’s gender attitude have not been studied in the Indian settings. The present study estimates the extent of covert modern contraceptive use (CCU) among women and its linkage with husbands’ gender attitudes in India.

**Methods:**

The study is based on fecund and monogamous couples using modern, reversible contraceptive methods. The numbers of such couples were 4,825 and 7,824 in the national family health surveys 2005–06 and 2015–16 respectively. The outcome variable in the study was CCU, while the independent variables were husband’s gender attitude, women’s education, freedom of mobility, freedom to spend money independently, surviving number of children, concordance regarding additional children, couple-level information such as age and educational gap between spouses, and some socioeconomic status (SES) variables. We used latent class analysis to measure the gender attitude and used bivariate descriptive analysis and multivariate binary logistic regression to assess the linkages between husband’s gender attitude and CCU.

**Results:**

This study found that the prevalence of CCU increased from 15% in 2005–06 to 27% in 2015–16. In both the time periods, contraceptive pills were the most preferred covert method, followed by intrauterine device (IUD). The results of the multivariate logistic regression show that women with husbands of moderate and low egalitarian gender attitudes were, respectively, 50% and 40% more likely to hide their contraceptive use than those with husbands of a high gender attitude. Women’s education, wealth index, number of living children, and region of residence were also found to be significantly associated with CCU.

**Conclusion:**

The study reveals that husband’s low egalitarian gender attitude can be a potential barrier between spouses, preventing them from opening up about their fertility preferences and contraceptive needs to each other. A couple-oriented approach to family planning is needed so that both members of a couple can satisfy their fertility desires and preferences eventually.

**Supplementary Information:**

The online version contains supplementary material available at 10.1186/s12889-022-12882-x.

## Background

Researchers across countries have raised the issue of differences in reproductive attitudes and preferences between husband and wife, which leads them to have dissimilar contraceptive needs. Covert contraceptive use by women or men is the result of such a situation. It is defined as the use of a contraceptive method without informing the spouse. In recent years, an increasingly high proportion of women from many parts of the world have been found using modern contraceptive methods without informing their partners. In 2010s, the prevalence of covert use of modern contraceptive methods ranged from 20 to 40% in Sub-Saharan African countries [1–4]. The practice of hiding family planning has been found to be common among married adolescent girls in Nigeria [4]. In India, only a few studies have explored this dimension and found that the prevalence of covert contraceptive use varies from 18 to 68% [5–7]. While the practice of CCU in India has been inadequately studied as we have not come across any nationwide systematic study that has attempted to link husband’s egalitarian gender attitude and CCU among Indian married women (age 15–49 years). Our study fills this knowledge gap in the Indian and similar sociocultural contexts.

### Gender attitude and contraceptive use

CCU also represents women’s own decision to practice contraception without the direct involvement of the husband. It is the result of a combination of spousal differences in fertility preferences or intentions and husband’s strong disapproval of contraception. In 1998, Biddlecom and Fapohunda found that husband’s pronatalism, problematic inter-spousal communication, and husband’s opposition to contraception had led to CCU for modern methods among women [8]. Studies from high fertility regions suggest that men do not approve of contraception since women are expected to bear children solely according to husband's fertility preferences [9, 10]. Husbands also disapprove of contraceptive use by wives out of the fear of losing control over their sexual behaviour [11]. In the past, men's egalitarian gender attitude has been found to be positively associated with fertility preferences and contraceptive use. Such attitude shapes an individual’s reproductive choices considering other partner’s desires and preferences [12]. Men's overall fertility goals and plans are significantly affected by their gender ideology [13]. Several studies established that men with lower egalitarian gender attitude had higher fertility aspirations [14–16] and such associations can potentially lead to low or covert contraceptive use [16]. In contrast, others find that more egalitarian men were likely to have higher fertility intentions than their traditional counterparts [17, 18].

### Women’s autonomy and contraceptive use

In a cultural setting like India, women’s reproductive preferences in general and freedom of contraceptive choice in particular are often compromised, and the major reason for such subjugations are often husband’s low egalitarian gender attitude and a systematic cultural disposition. Various forms of patriarchal oppression are vehemently pro-natal and likely to restrict women's freedom of reproductive choices [19]. In most male-dominated societies, husbands not only dominate decision-making in contraceptive use, birth spacing, sex-preference for children and timing of the next birth [20–23], but are also a major barrier to women’s autonomy and control over their lives [24]. In a recent Indian study, only 12% of women were found to make independent decisions regarding their healthcare; in the rest of the cases, the decisions were made by husbands either partially or solely [25]. Women’s dependency on their partners is particularly evident in curtailing their mobility and such a behaviour can potentially restrict them from replenishing and replacing the method in use or treating its side effects [26].

The issue of restricted mobility is widespread in India as a majority of women irrespective of their socioeconomic strata required permission to visit a health facility, visit friends and relatives, go shopping in the neighbourhood, or even going to short distances [27]. In a marital relationship, greater autonomy gives women the power to negotiate their reproductive choices to some extent and is found to be positively associated with better utilization of maternal healthcare and family planning services by women [28–32].

Apart from husband’s gender attitude and mobility, women’s educational attainment, working status, parity, religion, place of residence, discordance in desire number of children are found to influence the covert contraceptive use. In India, spousal schooling and age gaps might also affect CCU as women tend to marry more educated or older men than themselves and they possibly internalized husband’s high handedness in sexual and reproductive healthcare decisions, thinking the husband would make wiser decisions for them[1, 2, 33].

Only a few studies have so far associated the dimension of husband’s egalitarian gender attitude with wife’s covert use of modern reversible contraceptive methods, and none of them has been conducted in a highly socio-demographically diverse society like India. Such studies are especially relevant in the Indian society where men’s gender attitudes and cultural norms vary greatly and are yet to attain an egalitarian form along with gender equity in contraceptive use. Doing so becomes all the more essential as the patriarchal value system is still strong and men’s reproductive intentions and their decision-making in fertility preferences are supreme that can have profound consequences for women[27, 30]. Thus, the prime objective of this study is to estimate the extent of CCU and its correlates in the Indian context using a nationally representative sample of women. The present study primarily examines the hypothesis whether the low egalitarian gender attitude of husbands is positively associated with wife's covert use of modern reversible contraceptive methods. The findings of this study would be useful for increasing positive male participation, which is one of the key strategies of the national family planning program [34]. The findings will provide evidence for achieving the SDGs that aim for universal access to sexual and reproductive healthcare and gender equality in informed decision-making regarding sexual relations and reproductive health, including contraceptive use (Targets 3.7 and 5.6.1).

## Methods

### Data

This study primarily used the fourth round of National Family Health Survey (NFHS) that was conducted in 2015–16. However, to see changes over time in the level of covert use of modern methods of contraception, the third NFHS (2005–06) round was also analysed. The National Family Health Survey (NFHS) is a large-scale, multi-round survey, conducted in a representative sample of households throughout India. In the selected households, all the eligible women aged 15–49 years were interviewed. Eligible men aged 15–54 years were interviewed only from a fraction of households selected for men’s interview. In the fourth round, the mandate of the NFHS, for the first time, was also to provide a majority of health and demographic indicators at the district level along with state and national levels. However, to save the resources, the state module, comprising men’s interview schedule and Sections 8–11 of women’s interview schedule, was canvassed only in 15% of the total households sampled in the fourth round. In contrast, men’s and women’s interview schedules were canvassed in all the sampled households in NFHS-3 (2005–06) since the mandate was to generate only state and national level indicators. Both the rounds of NFHS (2005–06 and 2015–16) were cross-sectional in nature and provided independent samples of couples in the couple data files, which were analysed for this study [35, 36].

### Selection of couples

The present analysis estimated covert use of modern reversible contraceptive methods that include pills, intrauterine devices, injectables, and other modern contraceptive methods for females. The analysis excluded couples among whom women either did not report using any method or reported using traditional methods or female/male sterilization. The other categories excluded were couples among whom either the man reported having more than one wife or the woman reported an additional wife of the husband, or where a man’s last sexual partner was not his wife since the questions asked to men on their contraceptive use pertained only to their last sexual partner. The study is based on 4,825 and 7,824 fecund, monogamous couples from NFHS-3 (2005–06) and NFHS-4 (2015–16), respectively, who were not sterilized and were users of modern reversible contraceptive methods. A few cases in which women accepted using a contraceptive after the last coitus were dropped from the study.

### Generating sampling weights for couples

In the DHS survey, men and women from the sampled households were interviewed separately. The DHS program provides men and women data files separately, with individual and household sampling weights. A couples’ data file on matched partners is also available but without the sampling weights for the couples. This issue cannot be bypassed by applying women’s or men’s sampling weights as doing so creates a significant bias in the results. The solution is to develop sampling weights for the couples to make the results representative [37]. Accordingly, normalized couple sampling weights were generated for this study based on the methodology suggested by Becker & Kalamar in 2018 [37]. Another study done in the Indian context by applying couples’ level weights but did not use an appropriate method for the weights’ calculation [38].

### Dependent variable

Since NFHS-4 (2015–16) did not directly ask a woman whether ‘your husband/partner knows about your contraceptive use’, the estimate for this dependent variable was indirectly obtained as suggested by Gaska & Becker in 2018 [1]. The survey asked women a series of questions like: ‘Are you currently doing something, or using any method, to delay or avoid pregnancy?’ If the answer was ‘Yes’, then a subsequent question was asked: ‘Which method are you using?’ For men, the question was asked more specifically as: ‘The last time you had sex, did you or your partner use any method (other than a condom) to avoid or prevent a pregnancy?’. The term ‘other than a condom’ was used within parentheses since a question regarding the use of condom at the last coitus was asked to men in a previous section of the questionnaire.

The dependent variable, ‘covert contraceptive use’, henceforth may be called ‘CCU’, is defined as the use of a female modern contraceptive method for spacing as reported by a woman but not by her husband. By this definition, if a woman reports using any female contraceptive method and a man reports using condoms, it is considered a case of open contraceptive use. If husband and wife report different female modern methods, that too is regarded as open contraceptive use.

### Independent variables

Based on the literature, a list of independent variables regarded to exert a direct or an indirect influence on covert contraceptive use were included in the analysis. The independent variables were categorized into two parts. The first category included women’s background characteristics, women’s education, religion, caste, place of residence, and wealth [5, 33, 39] while the second category included couple-level variables. Religion, that was included in the first category, was divided into three categories: Hindu, Muslim, and Other. Caste of women was also taken as an independent variable and categorized as: Scheduled Caste/Scheduled Tribe (SC/ST), Other backward class (OBC), and Other. Place of residence was divided into rural and urban sub-categories. Women’s economic condition was categorized into household wealth quintiles as: poor, middle, and rich. Household wealth quintile was taken as a proxy for access to resources in making decisions in general and using contraceptive in particular.

In order to control the regional variation, we included region as an independent variable and categorized states and union territories into different regions based on their geographical location. We adopted the standard categorization as per the NFHS. This categorization, to a great extent, also takes into account sociocultural and demographic variation, marriage and reproduction related customs, women’s status and the overall governance in implementing family planning program that may affect CCU.

On the women’s autonomy front, a battery of independent variables was inserted. Among them, women’s education included the following categories: non-literate, primary, secondary, and higher education. Other independent variables included women’s working status, that is, whether a woman had money she could use independently, and women’s freedom of mobility to visit health facility and market places. The literature suggest that these variables affect women’s contraceptive use as well as the covert contraceptive use [21–23]. Both variables were dichotomous, with ‘yes’ and ‘no’ responses.

We also included three couple-level variables. The first one was age difference between husband and wife, which was divided into the following categories: wife is the same age as husband or older, husband is 1–3 years older, husband is 4–6 years older, and husband is much older, which included couples where husband was 6 or more years older than wife. The second variable was educational difference between spouses and was divided into three categories, namely ‘both are equally educated’, ‘wife is more educated’, and ‘husband is more educated’. Education and age gap were included with the reasoning that a spouse senior by age or education tends to dominate in decision-making regarding reproduction and family planning [40–42]. We included concordance on desire for more children as the third couple-level independent variable and divided it into two categories, namely ‘yes’ (indicating concordance) and ‘no’ (indicating absence of concordance).

Husband’s egalitarian gender attitude was taken as the main explanatory variable in this study. The working mechanism of this variable is unique and explained below in detail.

### Measurement of husband’s egalitarian gender attitude

Husband’s egalitarian gender attitude was measured from the responses to questions posed to men on their perception of various situations in NFHS-4. In the first battery of questions, husbands were asked if beating or hitting wife is justified in the following situations: a) if she goes out without telling husband b) if she neglects children c) if she argues with husband d) if she refuses to have sex with husband e) and if she doesn’t cook food properly. In the second battery, husbands were asked whether wife is justified in refusing sex if she knows: a) husband has a sexually transmitted infection (STI) b) if husband has sex with another woman c) and if wife is tired or not in the mood. In the third series of questions, husbands were asked if, in the situation that wife refuses sex, husband has the right to: a) get angry, b) refuse to give her money or other financial support, c) use force to have sex, d) go and have sex with another woman. The responses to each of these questions were coded as ‘0’ if the attitude was negative and ‘1’ if it was positive. This study used the Latent Class Analysis (LCA) to measure husband’s egalitarian gender attitude. LCA is a statistical procedure used to group individuals into classes of an unobserved variable based on responses made on a set of nominal or ordinal observed variables. It can identify subgroups of individuals who share common characteristics in such a way that they have a similar scoring pattern within the group [43]. The mathematical equation for LCA is as follows:

$${\varvec{P}}\left({\varvec{Y}}={\varvec{y}}\right)=\sum_{{\varvec{c}}=1}^{{\varvec{C}}}{{\varvec{\delta}}}_{{\varvec{c}}}\prod_{{\varvec{i}}=1}^{{\varvec{m}}}\prod_{{\varvec{j}}=1}^{{{\varvec{n}}}_{{\varvec{i}}}}{{\varvec{\tau}}}_{{\varvec{i}}{\varvec{j}}{\varvec{c}}}^{{\varvec{I}}({{\varvec{y}}}_{{\varvec{i}}}={\varvec{j}})}$$

where j = 1, 2, … n_i_ represents the number of possible categories the variable y_i_ can take and I(y_i_ = j) is the indicator function that equals to 1 if the response is y = j and, 0 otherwise. ${\varvec{\delta}}$ c is the probability of membership in the latent class c and the sum of probabilities for all classes equal to 1. ${{\varvec{\tau}}}_{{\varvec{i}}{\varvec{j}}{\varvec{c}}}^{{\varvec{I}}({{\varvec{y}}}_{{\varvec{i}}}={\varvec{j}})}$ is the probability of variable I taking j^th^ value in c^th^ class.

An appropriate number of classes (three) were selected on the basis of the Akaike Information Criterion (AIC) and the Bayesian Information Criterion (BIC). The AIC and BIC values of two-, three-, and four-class models is given in the appendix. The values of AIC and BIC were used to develop a three-class model, namely low egalitarian, moderately egalitarian, and highly egalitarian class. In order to arrive at an optimization for achieving the global maxima, up to 300 iterations were fixed in advance and a tolerance in the log likelihood of degree at 10^–7^ was considered for the convergence of the model. The maximum likelihood ratio test (Chi-square) was attained at the value of 22,783.15 with a p-value < 0.000 (Appendix Table A).

The ‘low egalitarian’ class included men who had a 27% to 56% more chance of justifying hitting or beating wife in five different scenarios and a 13%, 20%, and 24% chance, respectively, of believing that wife was not justified in refusing sex if she knew that the husband had an STI, if the husband had sex with another woman, and if the wife was tired or not in the mood. Men in this class also had the highest chance of giving ‘yes’ as the response to the question if the husband has the justification to get angry or reprimand his wife, refuse financial support, or have sex with another woman if the wife refused sex (Appendix Table B).

In the ‘moderately egalitarian’ class, the chance of justifying hitting or beating wife ranged from 2 to 10%. Moderately egalitarian men had a 67%, 94%, and 89% chance, respectively, of believing that the wife was not justified in refusing sex if she knew that the husband had an STI, if the husband had sex with another woman, and if the wife was not in the mood. The moderate class also included men who had a 2% to 5% chance of giving negative answers to the question on husband being justified in using force, or getting angry, or denying financial support to the wife if she refused to have sex with him (Appendix Table B).

The ‘highly egalitarian’ class included men who had the least chance of giving negative answers to all three batteries of questions. In this category, men had a 0.5% to 5.1% chance of justifying wife beating and a 1.5% to 5.6% chance of believing that the wife was not justified in refusing sex in different situations. They also had a 6% chance of giving ‘yes’ as the answer to the question that husband had the right to get angry if his wife refused sex. For the other questions in this section, they had less than 1% chance of having a negative attitude towards wife refusing sex (Appendix Table B).

For the analysis, bivariate and multivariate techniques were employed. The logistic regression analysis was carried out to understand which factors, among the ones included in NFHS-4 (2015–16), contributed to the covert use of modern reversible contraceptives. In the first model, we included husband’s gender egalitarian attitude variable only to estimate the unadjusted odds ratio of CCU, which gave the pseudo R^2^ value of 0.011. In the second model, we added couple-level variables and women’s socioeconomic characteristics also and the model produced the pseudo R^2^ value of 0.083, which shows an improvement over the first model (see chapter 5, Retherford and Choe, 1993, for details) [44].

Prior to the insertion of independent variables into our regression models, we checked the VIF to examine whether there was a multicollinearity among them [45]. The overall value of VIF was 1.4, while the values for the selected independent variables varied between 1.0 and 3.0. This is so because independent variables like place of residence, mobility, working status, wealth status, and freedom to use money by themselves seemed to be not so correlated with each other and with the core variable of interest, that is, husband’s egalitarian gender attitude.

## Results

**Fig. 1:**
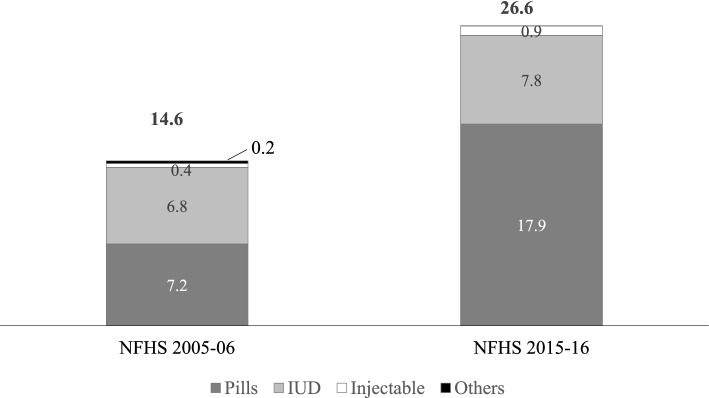
Percentage of women covertly using modern reversible contraceptive methods, 2005–2016

Figure 1 uncovers the changes that have occurred in the covert use of modern reversible contraceptives in the decade of inter-survey period. The percentage of covert use sharply increased from 14.6% in 2005–06 to 26.6% in 2015–16. In both the surveys, contraceptive pills were the preferred covert method for a majority of the women, followed by IUD and injectables. The contribution of contraceptive pills to all covert methods increased from 49.6% in 2005–06 to 67.2% in 2015–16. The contribution of injectables also increased. In contrast, the share of other modern contraceptives – which include diaphragm, foam, and jelly – which was already low in 2005–06 decreased further during the inter-survey period. In this context, it is necessary to note that the percentage increase in the use of pills, injectables, and condoms between 2005–06 and 2015–16 was 32.3%, 79.2%, and 7.7%, respectively, whereas the use of IUD decreased by 11.8% during the same period (data not shown here) [36, 37].

Table 1 gives the percentage of CCU by background characteristics. The covert use was more among those women whose husbands’ gender attitude was less egalitarian (30.6%) or moderately egalitarian (35.3%) than those whose husbands had a highly egalitarian gender attitude (23.4%). The prevalence of CCU was lower (18.3%) among practitioners of other religions than both Hindus (27.3%) and Muslims (26.1%). Nearly 30% of rural women were in CCU against only 22.1% of urban women. Working women or those who had worked within one year (30.6%) showed a higher level of CCU than their non-working counterparts (25.4%). CCU was found to be the highest among women who belonged to the poor wealth quintile, followed by those in the middle and rich quintiles. As many as 29.8% women who were more educated than their husbands reported CCU as against only 24.8% of those whose husbands were more educated than them. A large variation across regions was noticed in CCU. The Eastern region showed the maximum CCU level at 38.4%, while the minimum level was observed in the Northern region(15.6%).

**Table 1:** Percentage of women covertly using reversible modern contraceptive methods by background characteristics, 2015–16

Background characteristics	Number of modern contraceptive users	% of covert users	Chi-square statistics	p-value
**Husband’s gender attitude**				
Highly egalitarian	5299	23.4	102.7^***^	0.000
Moderately egalitarian	1047	35.3		
Low egalitarian	1478	30.6		
**Couple’s concordance regarding more children**				
Yes	6424	25.8		
No	1400	29.3	12.1^**^	0.001
**Religion**				
Hindu	5239	27.3		
Muslim	1533	26.1	7.2^*^	0.03
Other	1052	18.3		
**Caste**				
SC/ST	2391	28.6		
OBC	2570	26.6	78.2^***^	0.000
Other	2315	23.0		
**Place of residence**				
Rural	4823	29.6	57.1^***^	
Urban	3001	22.1		0.000
**Wife’s education**				
Non-literate	1439	26.7		
Primary	1009	31.1	96.2^***^	0.000
Secondary	4068	27.8		
Higher	1398	18.3		
**Age difference**				
Wife same age or older	584	23.6		
Husband 1–3 years older	2834	25.4	9.2^*^	0.027
Husband 4–5 years older	1830	26.3		
Husband 6 + years older	2576	27.7		
**Educational difference**				
No difference	1972	26.0		
Wife more educated	2181	29.8	2.9	0.237
Husband more educated	3671	24.8		
**Working status**				
No	6369	25.4		
Yes	1455	30.6	5.0^*^	0.026
**Wealth index**				
Poor	2163	36.4		
Middle	1528	27.6	235.7^***^	0.000
Rich	4133	20.6		
**Number of living children**				
0	279	8.0		
1	2277	26.4		
2	3077	27.8	57.4^***^	0.000
3 +	2191	27.2		
**Region**				
North	2663	15.6		
Northeast	1456	33.7		
East	1179	38.4	481.2^***^	0.000
West	564	28.7		
South	224	32.4		
Central	1738	17.1		
**Freedom to visit market**				
No	3202	27.5		
Yes	4622	25.6	12.7^***^	0.000
**Freedom to visit health facility**				
No	3521	26.8		
Yes	4303	26.0	15.7^***^	0.000
**Respondent has money to use alone**				
No	4119	28.0	11.1^**^	0.001
Yes	3705	24.9		

In terms of education, CCU was found to be the highest among women with primary education (31.1%) and the lowest among women with higher education (18.3%). More than one-fourth (27.5%) of women who did have freedom to visit market places were in CCU. The scenario was not very different among women who were allowed to visit the market (25.6%). Among women who had money which they could use for themselves, the prevalence of CCU was found at 24.9%, against 28.0% among their counterparts.

Both the variables of reproductive decision, that is, concordance regarding desire for more children and number of living children, showed little difference in CCU levels across the subgroups, except women with no living child, who had quite a low level (8.0%).

Table 2 reveals the results of the logistic regression analysis that provided the odds of covert contraceptive use (CCU) for selected background characteristics and husband’s gender egalitarian attitude. Two different regression models were applied. In the first model, husband’s gender attitude alone was used as the explanatory variable. In the second model, other background characteristics along with husband’s gender attitude variable were inserted. The unadjusted odds ratios unveiled the impact of husband’s gender egalitarian attitude towards CCU of modern spacing methods. The odds of CCU were 1.8 and 1.6 times higher among women whose husbands’ gender egalitarian attitude was moderate and low, respectively, compared with those women whose husbands had a highly egalitarian gender attitude. However, controlling other background characteristics in model 2, women having husbands with a moderately egalitarian and low egalitarian gender attitude were, respectively, 1.5 times [AOR: 1.54; 95%CI (1.32–1.80)] and 1.4 times [AOR: 1.40; 95%CI (1.21–1.60)] more likely to show CCU compared with women having husbands with a highly egalitarian gender attitude.

**Table 2:** Adjusted and unadjusted odds ratios for covertly using reversible modern contraceptive methods, 2015–16

**Sociodemographic characteristics**	Odds Ratio	P-value	95%CI		Odds Ratio	P-value	95%CI	
			Lower	Upper			Lower	Upper
**Husband’s gender attitude**								
High egalitarian								
Moderately egalitarian	1.84	0.00	1.6	2.13	1.54^***^	0.00	1.32	1.80
Low egalitarian	1.59	0.00	1.4	1.81	1.40^***^	0.00	1.21	1.60
**Couple’s concordance regarding more children**								
No								
Yes					0.89	0.11	0.76	1.00
**Religion**								
Hindu								
Muslim					1.01	0.89	1.06	1.41
Other					0.99	1.00	1.12	1.49
**Caste**								
SC/ST								
OBC					1.00	0.98	0.87	1.16
Other					0.78^***^	0.00	0.66	0.91
**Place of residence**								
Urban								
Rural					1.03	0.61	0.91	1.18
**Women’s education**								
Non-literate								
Primary					1.01	0.88	0.82	1.23
Secondary					0.89	0.24	0.75	1.07
Higher					0.68^***^	0.00	0.53	0.88
**Age difference**								
Wife same age or older								
Husband 1–3 years older					0.99	1.00	0.76	1.10
Husband 4–5 years older					1.03	0.77	0.8	1.30
Husband 6 + years older					0.97	0.82	0.76	1.21
**Educational difference**								
No difference								
Wife more educated					1.03	0.61	0.88	1.21
Husband more educated					0.92	0.21	0.79	1.05
**Working status**								
No								
Yes					1.11	1.44	0.92	1.16
**Wealth index**								
Poor								
Middle					0.95	0.60	0.81	1.12
Rich					0.78^*^	0.01	0.65	0.92
**Living children**								
0								
1					3.46^***^	0.00	2.76	3.96
2					3.57^***^	0.00	2.36	6.09
3 +					4.00^***^	0.00	2.63	6.97
**Region**								
North								
Northeast					2.6^***^	0.00	2.18	3.13
East					3.24^***^	0.00	2.76	3.95
West					1.97^***^	0.00	1.57	2.47
South					2.48^***^	0.00	1.84	3.47
Central					0.93	0.39	0.78	1.13
**Freedom to visit market place**								
No								
Yes					1.07	0.48	0.89	1.29
**Freedom to visit health facility**								
No								
Yes					0.81^*^	0.02	0.67	0.97
**Respondent has money to use alone**								
No								
Yes					1.06	0.31	0.95	1.20
Constant	0.29	0.00	0.28	0.32	0.08	0.00	0.04	0.15

^*^p < .05, *p < 0.01, ***p < .001, 95%CI – Confidence Interval at 95% level of significance

Caste, women’s education, wealth status, number of living children, region of residence, and freedom to visit a health facility were also found to be significantly associated with CCU. Women from the other caste subgroups were 22% [AOR: 0.78; 95%CI(0.66–0.91)] less likely to practice CCU than women belonging to the two socioeconomically disadvantaged groups. Similarly, women belonging to the rich stratum of wealth were 22% [AOR: 0.78; 95%CI(0.65–0.92)] less likely to show CCU than their counterparts from the poor wealth stratum. Regional variations in the chance of being a covert user of a modern spacing contraceptive method were quite visible. The odds of CCU were significantly higher among those women who resided in the Northeastern (2.6 times), Eastern (3.2 times), Western (2.0 times), and Southern (2.5 times) regions than in the Northern region.

Compared with no child, women with one or more child had at least 3.5 higher odds of being in CCU. Furthermore, women with a higher level of education were 32% [AOR: 0.68; 95CI% (0.53–0.88)] less likely to covertly use modern spacing methods of contraception than the non-literate women. Women who had freedom to visit a health facility were 19% less likely to report CCU of modern spacing methods than those who did not have such a freedom.

## Discussion

The present study indirectly estimated the covert contraceptive use (CCU) for modern reversible contraceptive methods and explored its association with husband’s egalitarian gender attitude and other socio-demographic characteristics. The prevalence of covert contraceptive use increased between NFHS-(2005–06) and NFHS-(2015–16). A possible explanation for this is the greater availability of contraceptive services in the recent years, which has made it easier for women to obtain contraception without their partner’s knowledge [1]. Wife’s concealment from husband of her modern reversible contraceptive use is also an indication of absence of communication, lack of confidence, or disagreement on fertility preferences and family planning. In many larger states in India, studies have observed an absence of communication among a significant proportion of couples [7, 46].

In India, contraceptive pills and IUDs are very popular among women as spacing methods, which is evident from the extent of CCU as well. Women pick IUDs and pills as CCU methods as they are easily available and can be used without husband’s consent. Contraceptive pills are affordable and accessible and can be easily hidden, although a husband can become suspicious and question his wife if her mensural cycle gets disturbed. This can also be the case with the covert use of IUD as women may experience bleeding or discomfort during the first few days of insertion or an irregularity in their menstrual cycle. In addition, IUD insertion is done in clinical settings and, therefore, requires travel time to reach the health facility and waiting time in receiving it. It also requires a mandatory follow-up during the initial days/months of insertion. However, once installed, IUD gives women no reason to worry for the next few years. In other countries, injectables contribute significantly to the covert contraceptive use [2]. In India, the share of injectables in CCU is trifling since their use is not so widespread and they are not as easily available as pills and IUDs.

The main aim of this study was to find the relationship between covert contraceptive use and husband’s egalitarian gender attitude, and the two were found to be associated. Wives of husbands with moderate and low egalitarian gender attitudes were more likely to hide their contraceptive use than those having husbands with a high egalitarian gender attitude. Men with a high egalitarian gender attitude had a more positive attitude towards wife’s sexual and reproductive rights, which allowed women to open up about their reproductive choices and preferences in the marital relationship. In contrast, husbands with moderate and low egalitarian gender attitudes had a traditional gender attitude towards women’s sexual and reproductive rights, which could have been a barrier to interspousal communication regarding contraception.

The low prevalence of CCU among wives of husbands with a low egalitarian gender attitude than wives of husbands with a moderate egalitarian gender attitude also reveals the threat of violence faced by women. This is because of the very high chance of a low egalitarian gender attitude man justifying wife beating in a given situation. In order to protect themselves from the risk of violence, women married to men with a low egalitarian gender attitude would rather not use any contraceptive than be a covert contraceptive user [46]. Husbands with a moderately egalitarian gender attitude too have higher chances of denying women their sexual rights, which becomes a motivation for women to hide their contraceptive use. Since such men have a lower chance of justifying wife beating compared to men with a low egalitarian gender attitude, it provides courage to women to fulfill their contracepting needs [47].

The gender attitude of individuals not only affects the contraceptive use directly, but it has also been found to be associated with high fertility aspirations among men, which ultimately lead to low contraceptive use [18, 48]. In addition, the gender attitude of men and women has proven to be a strong predictor of quality of married life, with men who adopt less traditional gender attitudes having a better perception of quality of marital life [49]. On the other hand, men’s negative gender attitude towards sexual rights of women and their use of violence against women are possible barriers to a quality relationship. If a woman struggles for a healthy marital relationship, it is obvious that she will not get her basic sexual and reproductive rights effortlessly. Concealment of contraceptive use from the spouse is an easy way for such women to fulfil their needs for family planning. Such a concealment, whether in Indian or similar settings, poses challenges because families are a close-knit network in which a woman needs to hide her contraceptive use not only from her husband but also from her mother-in-law and other women co-residents. In circumstances where community health workers are well connected with the husband and the other co-residents, they may also reveal the woman’s contraceptive status unintentionally. Such possibilities expose women to the risk of domestic violence and to the continuity of their covert use of contraception.

Like other studies, our study also discovered a more open contraceptive use among couples where wife was highly educated. This shows that education gives more power to a woman in a marital relationship, allowing her to negotiate her reproductive choices [8, 50, 51]. The odds of concealment of contraceptive use were found to be low among ‘Other’ caste groups that represent the higher strata in the Indian society. This was expected as upper caste women have been found to have more reproductive health and sexual rights, including contraceptive use, birth interval, and age at marriage, in the other studies as well [52]. A similar explanation rationalizes the lower odds of covert contraceptive use in the rich wealth quintile.

In contrast to another study in a similar setting [2], couple’s concordance on preferred number of children was not found to be associated with CCU in the present study. This could itself be linked to the measurement of concordance on preferred number of children among spouses. In the absence of interpersonal communication between spouses on each other’s fertility preferences, wife assumes that her and her husband’s fertility ideals and preferences are same, when in reality they can be dissimilar [46].

This paper also looked into the association between women’s autonomous movement and the concealment of contraceptive use from their husbands since the existing research reveals the relationship between women’s freedom of mobility and contraceptive use [31, 32]. Women who were not allowed to go to health facilities alone were more likely to keep their contraceptive use secret from husbands. Non-autonomous movement of women embodies their subordinate position in the marital relationship, in which a rational negotiation of their reproductive rights seems highly restricted. Maybe women who live a more subordinated life find concealment of contraceptive use to be the easiest way to make their reproductive choices. At the same time, their fear of disclosure can compel them to compromise on the quality of the contraceptives used. Women in such situations can face a delay in receiving the services since they are obligated to be accompanied by the husband or some other family member, which can result in an unintended pregnancy. Concealment of contraceptive use can produce major penalties for women. It can put them at the risk of intimate partner violence (IPV) due to the covert contraceptive use and, in some cases, put their very union at stake [6, 53–56]. On the contrary, the exposure to IPV and sexual and reproductive coercion itself can be a reason for covert contraceptive use among women, particularly in societies with low women empowerment [4].

Covert contraceptive use can also be low in societies in which women’s independent access to modern spacing contraceptive methods is limited possibly due to several programmatic factors. It may include excess focus on female sterilization, higher stigma among women in obtaining methods from sources other than female frontline workers, and poor quality of care in general [57, 58]. All these may be some of the reasons why this study found a relatively low covert use of modern reversible contraceptive methods in the northern region compared with the other regions in the country.

## Conclusion

This study shows the high and increasing trend of covert use of modern reversible contraceptive methods among Indian women. The study reveals that husband’s low egalitarian gender attitude can potentially prevent spouses from opening up about their fertility preferences and contraceptive needs to each other. Clandestine use may be the best way to protect women’s reproductive rights, but it also shows the lack of men’s involvement in reproductive health and behaviour. As per the ICPD programme of action, the aim of family planning programs must be to enable couples and individuals to decide freely and responsibly the number and spacing of their children [59]. We foresee that the results of our study will help policy makers and program managers in improving nationwide free use of reversible modern contraceptive methods rather than focusing on the female sterilization program. Male involvement in family planning is one of the key strategies in the program *Mission Parivar Vikaas,* that is, mission for the development of family, launched by the Government of India in 145 aspirational districts lagging behind in the level of contraceptive use. It is imperative for such strategies to have gender sensitization counselling sessions for couples instead of having them only for men or women. Moreover, in light of our findings, husbands of non-literate women, poor couples, and couples belonging to disadvantaged castes/tribes must be prioritized when implementing these strategies. These initiatives will expedite India’s attainment of the sustainable development goals 3 and 5.

### Limitations

The main strength of the study is the development of an index for husband’s egalitarian gender attitude and the establishment of its association with CCU of modern reversible methods. Despite this important strength, this study also has certain limitations. Firstly, various prior studies have shown that interspousal communication on family planning affects covert contraceptive use, but unfortunately NFHS does not collect information on this aspect. Similarly, the association between IPV and covert contraceptive use of reversible methods could not be explored because the sequence of the two events (the time of occurrence of IPV and the initiation of the contraceptive method) was not available in the dataset. The association between the two could have been spurious without having such a sequence, especially when the literature suggests that one can precede the another. We also caution readers that at least in a few cases, the husbands may have given socially desirable answers to the questions on gender attitude in the survey to avoid coming across as men with a low egalitarian gender attitude in front of the interviewers. Finally, we recommend adding a few questions in the next NFHS round on household chores and childcare responsibilities to further refine the measurement of husband’s egalitarian gender attitude.

## Data

Data used in this study is available in the public domain and can be obtained from the Demographic Health Survey website at https://dhsprogram.com/data/available-datasets.cfm

## Ethical approval

A separate ethical approval for this manuscript was not required as the national nodal agency for conducting all the rounds of the NFHS had already obtained the ethical clearance from the Institutional Ethical Review Board [34, 35]. The data used for this study is available at https://dhsprogram.com/data/available-datasets.cfm for public use.

## Supplementary Information

Below is the link to the electronic supplementary material.

**Additional file 1: Table  A** Model selection and specifications **Table B **Percentage of participants gave negative response to item and probability of giving negative response to each item in each class from three class Latent Class Analysis model.

## Abbreviations

**CCU**: Covert contraceptive use
SDGs: Sustainable development goals
IUD: Intrauterine device
AIC: Akaike information criterion
BIC: Bayesian information criterion
LCA: Latent class analysis
STI: Sexually transmitted disease
DHS: Demographic Health Survey
NFHS: National Family Health Survey
SC: Scheduled caste
ST: Scheduled tribe
OBC: Other backward class
ICPD: International Conference on Population and Development
