# Supplementary material for: Covert use of reversible contraceptive methods and its association with husband’s egalitarian gender attitude in India
Source: BMC Public Health. 2022 Mar 7;22:460. doi: 10.1186/s12889-022-12882-x (PMC8903557; doi:10.1186/s12889-022-12882-x)
Supplement: Supplementary file 1 — Additional file 1: Table A Model selection and specifications Table B Percentage of participants gave negative response to item and probability of giving negative response to each item in each class from three class Latent Class Analysis model. [file 12889_2022_12882_MOESM1_ESM.docx]

**Appendix**

Table A: Model selection and specifications

| **Models** | **AIC** | **BIC** |
| --- | --- | --- |
| **Two class model** | 212573.6 | 212778.2 |
| **Three class model** | 194379.7 | 194690.6 |
| **Four class model** | 203358.1 | 203775.4 |
|  |  |  |
| **Fit Statistic for three class model** | Model vs Saturated | |
| **Likelihood Ratio Chi2**  **P-value (<)** |  | 22783.15  0.000 |

Table B: Percentage of participants gave negative response to item and probability of giving negative response to each item in each class from three class Latent Class Analysis model.

| **Items** | **% Negative attitude** | **Three class LCA model** | | |
| --- | --- | --- | --- | --- |
|  |  | High | Moderate | Low |
| **Justified beating wife if** |  |  |  |  |
| Wife goes out without telling husband | 10.2 | 0.022 | 0.069 | 0.472 |
| Wife neglect child | 12.4 | 0.031 | 0.094 | 0.552 |
| Wife argues with husband | 14.5 | 0.051 | 0.107 | 0.560 |
| Wife refuse to have sex | 4.8 | 0.005 | 0.027 | 0.270 |
| Wife does not cook food properly | 5.7 | 0.006 | 0.046 | 0.314 |
| **Wife Justified refusing sex if** |  |  |  |  |
| Husband had STI | 12.4 | 0.015 | 0.671 | 0.134 |
| Husband has other women | 18.7 | 0.035 | 0.939 | 0.198 |
| Wife is tired not in mood | 19.8 | 0.056 | 0.893 | 0.236 |
| **If wife refuse to sex** |  |  |  |  |
| Husband has right to get angry | 13.6 | 0.060 | 0.049 | 0.493 |
| Husband has right to refuse to financial support | 8.0 | 0.006 | 0.020 | 0.369 |
| Husband has right to have force sex | 7.3 | 0.004 | 0.019 | 0.349 |
| Husband has right to have sex with another women | 7.3 | 0.006 | 0.019 | 0.322 |
| **LC Mean** |  | **0.627** | **0.149** | **0.224** |
